# Detailed investigation of B cell populations following vaccination and infection with severe acute respiratory syndrome coronavirus-2 during pregnancy

**DOI:** 10.3389/fimmu.2026.1849532

**Published:** 2026-06-16

**Authors:** Laura Scholz, Nils Hoymann, Suzan Alboradi, Valeriia Grabar, Gina Marie Uehre, George Toth, József Mészáros, Paolo Gennari, Svetlana Tchaikovski, Atanas Ignatov, Mandy Busse

**Affiliations:** 1 Experimental Obstetrics and Gynecology, Medical Faculty, Otto-von-Guericke University, Magdeburg, Germany; 2 University Hospital for Obstetrics and Gynecology, Medical Faculty, Otto-von-Guericke University, Magdeburg, Germany; 3 University Clinic for Obstetrics and Gynecology, Medical Faculty, Brandenburg Medical School Theodor Fontane, Brandenburg an der Havel, Germany

**Keywords:** B cells, COVID-19, pregnancy, SARS-CoV-2, vaccination

## Abstract

**Background:**

Pregnancy induces significant immunological adaptation, including shifts in the balance between B effector and B regulatory cells. However, the impact of SARS-CoV-2 infection or vaccination on B cell populations during pregnancy remains largely unexplored.

**Methods:**

Blood samples were collected from 139 women prior delivery and grouped according to the questionnaire responses and serology: controls (uninfected/unvaccinated); previously infected only; vaccinated only; both vaccinated and infected; and acutely SARS-CoV-2 infected (unvaccinated or vaccinated). Maternal serum cytokine levels were determined, and B cell populations were analyzed by flow cytometry following short- and long-term stimulation with CpG ± CD40L and PMA/ionomycin or.

**Results:**

Serum levels of APRIL, IL-4, IL-6, TNF-α and sCD40L varied according to SARS-CoV-2 vaccination and infection status. Vaccination against SARS-CoV-2, and to a lesser extent infection with the virus, altered the frequency of various B cell populations, including plasma blasts, plasma cells and B memory cells. Patients infected with the virus exhibited increased levels of IL-10+ B cells, and decreased levels of IL-6+ B cells, in comparison to vaccinated women. In addition, the expression of CD40 was induced in B cells in response to infection. Conversely, the expression of PD-1, FasL and CD86 was enhanced by vaccination.

**Conclusion:**

SARS-CoV-2 infection and vaccination during pregnancy considerably shift the balance between pro- and anti-inflammatory B cell populations, and modified expression of costimulatory molecules. This highlights the need for further investigation into the long-term consequences of maternal SARS-CoV-2 immunity for both mothers and offspring.

## 1 Introduction

The complexity of the maternal immune system is characterized by its capacity to impede an immune response against a semi-allogenic fetus, while concurrently protecting the mother from harmful pathogens. However, these immunological adaptations may also render pregnant women vulnerable to adverse complications from respiratory viral infections.

Despite the official end of the SARS-CoV-2 pandemic, coronavirus infections are expected to occur annually, exhibiting seasonal tendencies (1). The present focus is on the long-term consequences of infection or vaccination with SARS-CoV-2, the full extent of which is only gradually becoming apparent. The hallmark of Long-COVID is persistent inflammation, accompanied by endothelial damage, T cell exhaustion, and alterations in B cell populations, including memory cells and antibody-producing cells (2, 3). It has been demonstrated that a multitude of autoantibody-producing B cells have been identified in cases of Long-COVID (4), analogous observations have been made in cases of post-vaccine syndrome (5, 6). The etiology of these syndromes remains an area of research with much to be explored. The available data are limited, particularly with regard to infections or vaccinations during pregnancy. A cost-benefit analysis is therefore critical for identifying risk groups with a high probability of a severe course of the disease and for the subsequent recommendation of annual vaccination against the predominant viral variant. This is the only way to reduce morbidity and mortality. Particular emphasis should be placed on pregnant women, who are at elevated risk of complications from respiratory viral infections. While vaccination against influenza viruses during pregnancy has been established as effective in preventing pregnancy complications for many years (7), the issue of general vaccination against coronaviruses during pregnancy remains incompletely clarified.

Therefore, it is imperative to evaluate the potency and durability of the immune response against SARS-CoV-2 in pregnant women following infection or vaccination. Antibodies serve as a key indicator of past SARS-CoV-2 infection or vaccination. The regulatory processes governing antibody production are closely linked to the generation and maintenance of plasmablasts and plasma cells from B cell progenitors. Antibody-secreting cells are defined as terminally differentiated cells arising from activated B cells that have previously undergone germinal center reactions (8). Here, short-lived plasmablasts are rapidly produced and provide early antibody response, whilst long-lived plasma cells sustain enduring humoral immunity (8). Recently, we demonstrated that the presence of antibodies against the SARS-CoV-2 spike or nucleocapsid protein in maternal blood depends on vaccination status or previous infection, and correlate with antibody levels observed in cord blood (9).

B cells play a pivotal role in healthy pregnancies due to their capacity to produce asymmetric antibodies and facilitate maternal-fetal tolerance, a process that is particularly facilitated by B regulatory (Breg) cells (10, 11). Breg cells maintain immune tolerance and homeostasis through several mechanisms, most notably via secretion of IL-10 (12). Multiple human B cell subsets can produce IL-10, including CD24^hi^CD38^hi^, CD24^hi^CD27+ and CD1d^hi^CD5+ B cells (13–16). These B cell populations exhibit not only divergent phenotypes but also disparate functional activities.

For many years, our research has focused on the role of B cells in healthy pregnancy and pregnancy-associated disorders. Nevertheless, the characterization of B cell subsets and the generation of antibodies during natural infection and/or vaccination remains incomplete in pregnancy, underscoring the need for further research in this domain.

The objective of the present investigation was to enhance the comprehension of the immunological consequences of vaccination and/or infection during pregnancy, with a particular emphasis on alterations in B cell populations in women at term. Our present study should constitute the basis for further research concerning the shift in the balance between regulatory B cells and effector B cells, including autoreactive B cells.

## 2 Materials and methods

### 2.1 Human subjects

Patients included in this study were recruited for our biobank between March 2022 and February 2024, which was approved by the ethics committee of the Otto-von-Guericke University medical faculty (EK19/22). Informed consent was obtained from all participants prior to their involvement in the study. The demographic data of the patients are summarized in **Table 1**. A total of 40 ml of venous blood was collected from each pregnant woman upon admission to the delivery room. Concurrently, both a SARS-CoV-2 rapid test and a polymerase chain reaction (PCR) test of nasopharyngeal swabs were performed. All procedures were performed in accordance with the established protocols. The blood was processed within 1h of collection, and peripheral blood mononuclear cells (PBMCs) were separated by Ficoll-density gradient centrifugation. PBMCs were washed twice with PBS, resuspended in freezing medium (10%DMSO+ 90% FBS) and stored in liquid nitrogen until use.

**Table 1:** Study cohort.

Characteristics	unit	Control (N=13)	Vaccinated /not infected (N=32)	Infected/not vaccinated (N=23)	Infected/ vaccinated (N=56)	Acute infected/ not vaccinated (N=5)	Acute infected/ vaccinated (N=10)	*p*
maternal								
age	years	29.2 ± 6.6	31.4 ± 4.8	30.1 ± 6.4	32.0 ± 6.4	32.2 ± 7.4	30.7 ± 3.2	0.6438
gestational age	weeks	38.3 ± 1.7	39.6 ± 1.4	38.7 ± 1.5	38.7 ± 2.2	40.0 ± 0.7	39.0 ± 1.3	0.0466
birth mode	vaginal birth	7 (54%)	20 (62%)	15 (65%)	22 (39%)	2 (40%)	6 (60%)	0.0455
	cesearean section	6 (46%)	12 (38%)	8 (35%)	34 (61%)	3 (60%)	4 (40%)	
gravida		3.2 ± 2.1	2.2 ± 1.5	2.8 ± 1.9	2.6 ± 1.8	3.2 ± 2.2	1.9 ± 1.0	0.2444
para		2.7 ± 2.1	1.8 ± 1.0	2.1 ± 1.3	1.9 ± 1.0	2.6 ± 1.5	1.6 ± 0.8	0.3334
blood loss	ml	358 ± 128	338 ± 120	352 ± 172	421 ± 172	300 ± 100	535 ± 355	0.0752
Hb (pre partal)	mmol/l	7.2 ± 1.2	7.5 ± 0.7	7.3 ± 0.5	7.5 ± 0.7	7.4 ± 1.0	7.7 ± 0.7	0.6993
Hb (post partal)	mmol/l	6.6 ± 1.1	6.8 ± 0.7	6.8 ± 0.9	6.7 ± 0.8	6.9 ± 1.5	6.8 ± 0.7	0.9896
neonatal								
birth weigth	g	3282 ± 584	3498 ± 410	3225 ± 443	3484 ± 500	3688 ± 474	3520 ± 706	0.1291
body length	cm	49.7 ± 2.7	51.6 ± 2.2	50.7 ± 2.2	51.2 ± 3.6	53.6 ± 1.8	52.2 ± 2.3	0.0274
APGAR (1 min.)		9.2 ± 0.6	9.0 ± 1.0	9.0 ± 0.8	8.6 ± 1.5	9.2 ± 0.4	9.2 ± 0.4	0.4273
APGAR (5 min.)		9.7 ± 0.6	9.3 ± 1.5	9.6 ± 0.7	9.4 ± 1.2	9.6 ± 0.5	10.0 ± 0.0	0.3094
APGAR (10 min.)		9.9 ± 0.3	9.7 ± 0.6	9.9 ± 0.4	9.7 ± 0.6	10.0 ± 0.0	10.0 ± 0.0	0.2108
CB pH		7.3 ± 0.09	7.3 ± 0.09	7.3 ± 0.06	7.3 ± 0.09	7.2 ± 0.10	7.3 ± 0.06	0.4382
CB base excess	mmol/l	-0.9 ± 4.4	-1.8 ± 4.2	-1.4 ± 2.6	-1.8 ± 3.6	-2.1 ± 4.3	-2.8 ± 2.8	0.5984
SARS-CoV-2								
infection (trimester)		0	0	2.7 ± 1.3	2.8 ± 1.3	4 ± 0	4 ± 0	<0.0001
SARS-CoV-2 ct value at birth		0	0	0	0	28.1 ± 10.8	26.4 ± 8.9	<0.0001
last vaccination (trimester)		0	1.6 ± 0.6	0	1.7 ± 1.3	0	1.1 ± 0.3	<0.0001
vaccination	number	0	2.5 ± 0.7	0	2.3 ± 0.7	0	2.4 ± 0.7	<0.0001

The study cohort comprised 13 women without SARS-CoV-2 infection or vaccination (control), 32 vaccinated but uninfected women, 23 unvaccinated but infected women, and 56 women who were both infected and vaccinated. Furthermore, the study incorporated a total of fifteen women diagnosed with acute SARS-CoV-2 infections. Of these subjects, five had not received any prior vaccinations, while ten had received at least one dose of a vaccine. The maternal characteristics encompassed a range of factors, including maternal age, gestational age, mode of delivery, number of pregnancies and parities, blood loss, and pre- and postpartum hemoglobin (Hb) values. The neonatal characteristics encompassed the following metrics: birth weight (in grams, g), birth length (in centimeters, cm), APGAR scores at one minute, five minutes, and ten minutes after birth, cord blood pH value, and base excess (in millimoles per liter, mmol/L). The parameters of the study included the trimester in which the infection was diagnosed (0, no infection; 1, up to the end of the 12th week of pregnancy; 2, 13th to the end of the 28th week of pregnancy; 3, 29th to the end of the 41st week of pregnancy (no SARS-CoV-2 detected at delivery); 4, SARS-CoV-2 detected at delivery) and the SARS-CoV-2 ct value at birth. Furthermore, the trimester of the final vaccination dose and the total number of vaccinations administered were documented. The subsequent analysis of the data was conducted using the Shapiro-Wilk test, followed by One-way ANOVA or Kruskal-Wallis test, as appropriate. Fisher's exact test was utilized in order to ascertain alterations in birth mode.

### 2.2 Stimulation of PBMCs

PBMCs were thawed and cultured in RPMI1640 supplemented with 1% penicillin/streptomycin and 10% fetal bovine serum (FBS) at 37 °C and 5% CO_2_ overnight. Subsequently, a cell count was performed using the Attune NxT counting function, after which the cells were either directly stained or stimulated for a period of either 5h (short-term stimulation) or 72h (long-term stimulation). For short-term stimulation, 2x10^6^ PBMCs/ml were stimulated with Phorbol 12-myristate 13-acetate (PMA; 50ng/ml) and ionomycin (500 ng/ml; both Sigma Aldrich, Darmstadt, Germany) in the presence of Brefeldin A (Biolegend; San Diego, USA) for 5h at 37 °C and 5% CO_2_. For long-term stimulation, PBMC were also stimulated with CpG ODN2006 (10μg/ml; Invivogen; San Diego, USA) alone or combined with human CD40L (1μg/ml; R&D systems; Minneapolis, USA) for 72h at 37 °C and 5% CO_2_. PMA (50ng/ml), ionomycin (500 ng/ml) and Brefeldin A were added for the final 5h of culture. Following the stimulation process, the PBMCs were harvested, thoroughly washed twice with FACS staining buffer, and subsequently distributed to the appropriate staining panels.

### 2.3 Cell staining and flow cytometry

3x10^5^ PBMCs were subjected to staining for cell surface markers for 30 min at 4 °C (see **Supplementary Table 1** for antibody details). For intracellular protein analysis, cells were fixed for 30 min with Fix&Perm solution, followed by staining with the appropriate antibodies (**Supplementary Table 1**); all reagents ThermoFisher/Ebioscience, San Diego, USA or Miltenyi Biotech, Bergisch Gladbach, Germany). In order to ensure the accurate gating of cell populations, the use of Fluorescence Minus One (FMO) controls for each antibody was employed. Measurements were performed on an Attune NxT flow cytometer (ThermoFisher Scientific, Waltham, USA) and analyzed with FlowJo software (Ashland, USA).

### 2.4 Detection of B cell associated mediators in serum samples

B cell-associated mediators in maternal serum were quantified using the LEGENDplex human B cell panel from BioLegend (San Diego, USA) following the manufacturer’s protocol.

### 2.5 Data analysis and statistics

Statistical analysis was performed using GraphPad Prism 8.0 software. Normality of distribution was determined by Shapiro-Wilk test. Data were analyzed by student´s t-test/Mann-Whitney-U test or Two-way ANOVA/Kruskal-Wallis test followed by Dunnett´s or Dunn’s multiple comparison test wa*s* used. Matched data were analyzed by Friedman test, followed by Dunn´s multiple comparison test. Correlation analyses were performed using the Spearman correlation test. *p<0.05; **p<0.01; ***p<0.001; ****p<0.0001.

## 3 Results

### 3.1 Study cohort

The study comprised 13 unvaccinated and uninfected with SARS-CoV-2 controls, 32 vaccinated but uninfected patients, 23 unvaccinated but infected patients, 56 vaccinated and infected patients, and 15 pregnant women in the acute phase of SARS-CoV-2 infection (5 unvaccinated and 10 vaccinated) (**Table 1**). The data were obtained via questionnaire and confirmed by anti-spike and anti-NCP antibody detection in maternal blood (9). Minor differences between the groups were observed in gestational age at delivery, newborn body length, and mode of delivery. Most infections with SARS-CoV-2 during pregnancy occurred during the second and third trimesters, whereas most vaccinations were administered during the first and second trimesters, with a mean of two to three doses (**Table 1**).

### 3.2 Acute SARS-CoV-2 infected patients differ in their immune response according to vaccination status

B cell-associated mediators in maternal serum were measured (**Table 2**). The levels of IL-6 were elevated in acutely infected and vaccinated pregnant women compared to the control group, while the APRIL levels were reduced in infected, unvaccinated patients. Between vaccinated/uninfected and infected/unvaccinated women, we detected a significant difference in the APRIL level (p=0.0036) as well as a positive correlation between IL-6 and APRIL with BAFF. Except for patients with acute infection, IL-6 correlated positively with IFN-γ, TNF-α and IL-10, whereas IL-10 also correlated positively with IFN-γ and TNF-α (**Supplementary Table 2A**). Comparison between acute SARS-CoV-2-infected patient groups revealed that unvaccinated pregnant women had significantly lower serum levels of IL-4, IL-6 and TNF-α, and higher serum levels of sCD40L (**Table 2**).

**Table 2:** Maternal serum cytokines.

Cytokine	Control	Vaccinated /not infected	Infected/ not vaccinated	Infected/ vaccinated	Acute infected/ not vaccinated	Acute infected/ vaccinated	p
IL-2	64.7 ± 21.6	73.7 ± 40.7	69.6 ± 31.8	66.6 ± 25.0	54.4 ± 22.3	107.0 ± 85.8	0.5326
IL-4	107.6 ± 47.1	137.8 ± 99.7	166.2 ± 181.1	119.2 ± 81.0	73.4 ± 8.2	197.7 ± 190.5	0.1924
IL-6	39.3 ± 21.0	44.5 ± 37.2	33.9 ± 19.4	38.8 ± 24.2	30.7 ± 6.8	74.9 ± 54.5	0.0489
IL-10	18.1 ± 5.2	20.9 ± 10.5	19.1 ± 11.9	19.0 ± 13.2	14.0 ± 4.5	33.0 ± 28.5	0.2827
IL-13	10.7 ± 2.8	12.4 ± 4.7	11.4 ± 3.5	11.6 ± 3.6	10.7 ± 3.7	22.3 ± 28.5	0.9145
TNF-α	76.0 ± 49.8	72.8 ± 43.4	74.0 ± 45.2	65.5 ± 38.6	43.2 ± 11.9	96.9 ± 75.9	0.2737
APRIL	20279 ± 11470	20945 ± 9057	14370 ± 6525	19654 ± 11965	18883 ± 5343	20566 ± 14921	0.0499
BAFF	2228 ± 1557	1790 ± 1511	1434 ± 804	1584 ± 834	1784 ± 637	2125 ± 1219	0.4274
CD40L	14353 ± 11196	10454 ± 6246	10314 ± 9503	11211 ± 6420	15413 ± 5082	8055 ± 5577	0.2470
IFN-γ	112.6 ± 63.8	110.7 ± 96.8	107.6 ± 58.8	94.0 ± 70.6	65.7 ± 19.8	102.9 ± 62,9	0.2334
IL-17A	18.4 ± 10.3	19.0 ± 12.0	15.6 ± 6.4	15.9 ± 7.5	10.8 ± 2.7	17.3 ± 7.7	0.1338
IL-12p70	33.5 ± 12.1	34.7 ± 16.6	31.5 ± 14.0	31.3 ± 13.3	23.0 ± 11.8	27.1 ± 12.9	0.2923
TGF-β	142.2 ± 81.6	163.9 ± 96.2	165.0 ± 94.3	147.4 ± 85.6	74.4 ± 14.1	144.8 ± 133.2	0.1465

Maternal serum (N = 13 controls, 32 vaccinated/ not infected, 23 infected/ not vaccinated, 56 infected/ vaccinated, 5 acute infected/ not vaccinated and 10 acute infected/ vaccinated patients) was taken immediately before delivery. Cytokine levels were determined using a Legendplex multiplex bead-based assay. Data were analyzed by Shapiro-Wilk test, followed by Kruskal-Wallis test. Shown are the mean and SD values.

### 3.3 Alterations of B cell populations following SARS-CoV-2 infection or vaccination

**Figure 1:**
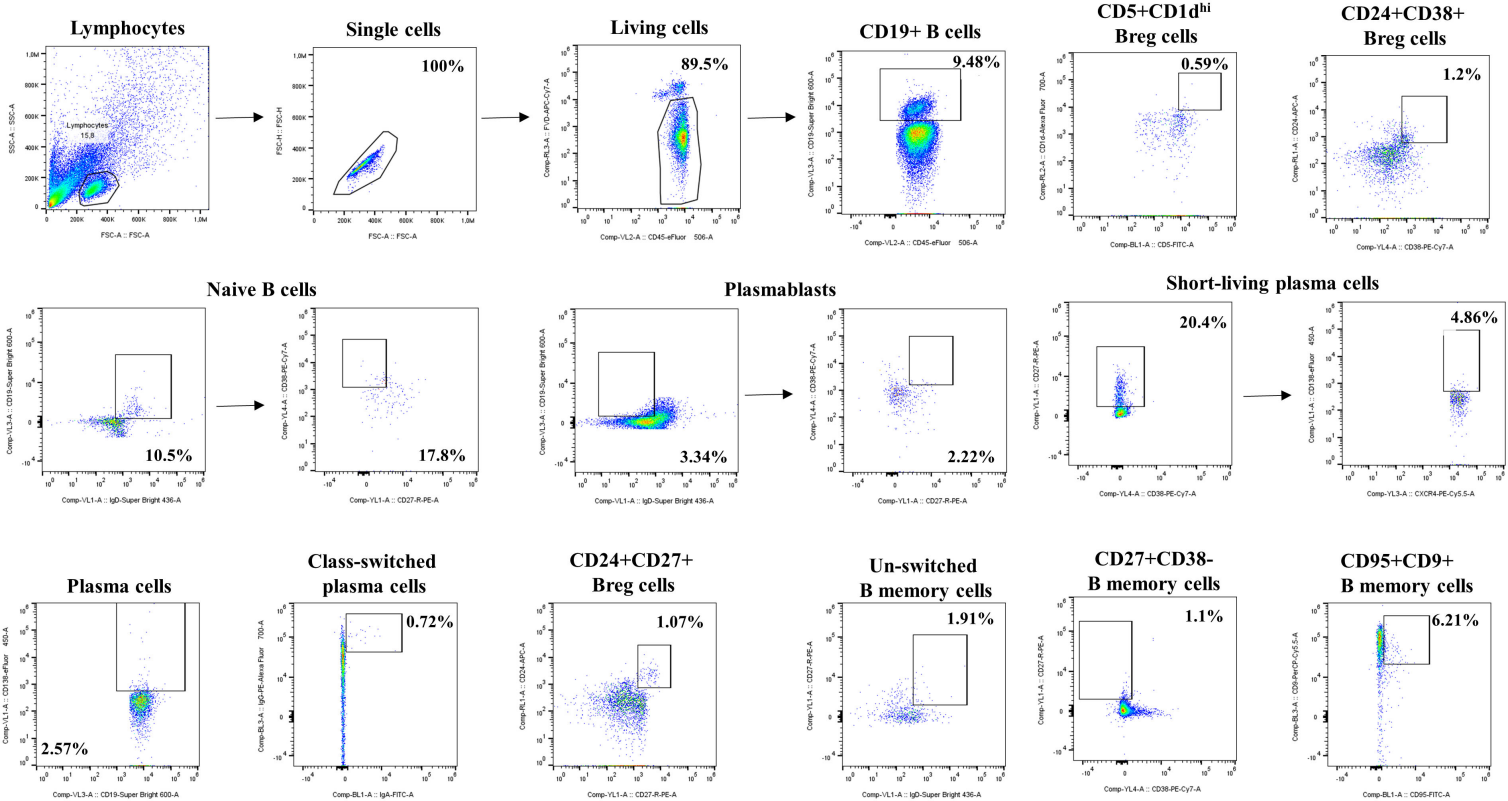
The image illustrates the flow cytometry gating strategy. The analysis was conducted on the basis of the following gating strategy: lymphocytes, single cells, living cells, and CD19+ B cells. Then naïve B cells, Breg cell populations, plasmablasts, short-living plasma cells, plasma cells, class-switched plasma cells, un-switched B memory cells and memory cells. The configuration of all gates was conducted in accordance with minus 1 controls.

**Figure 2:**
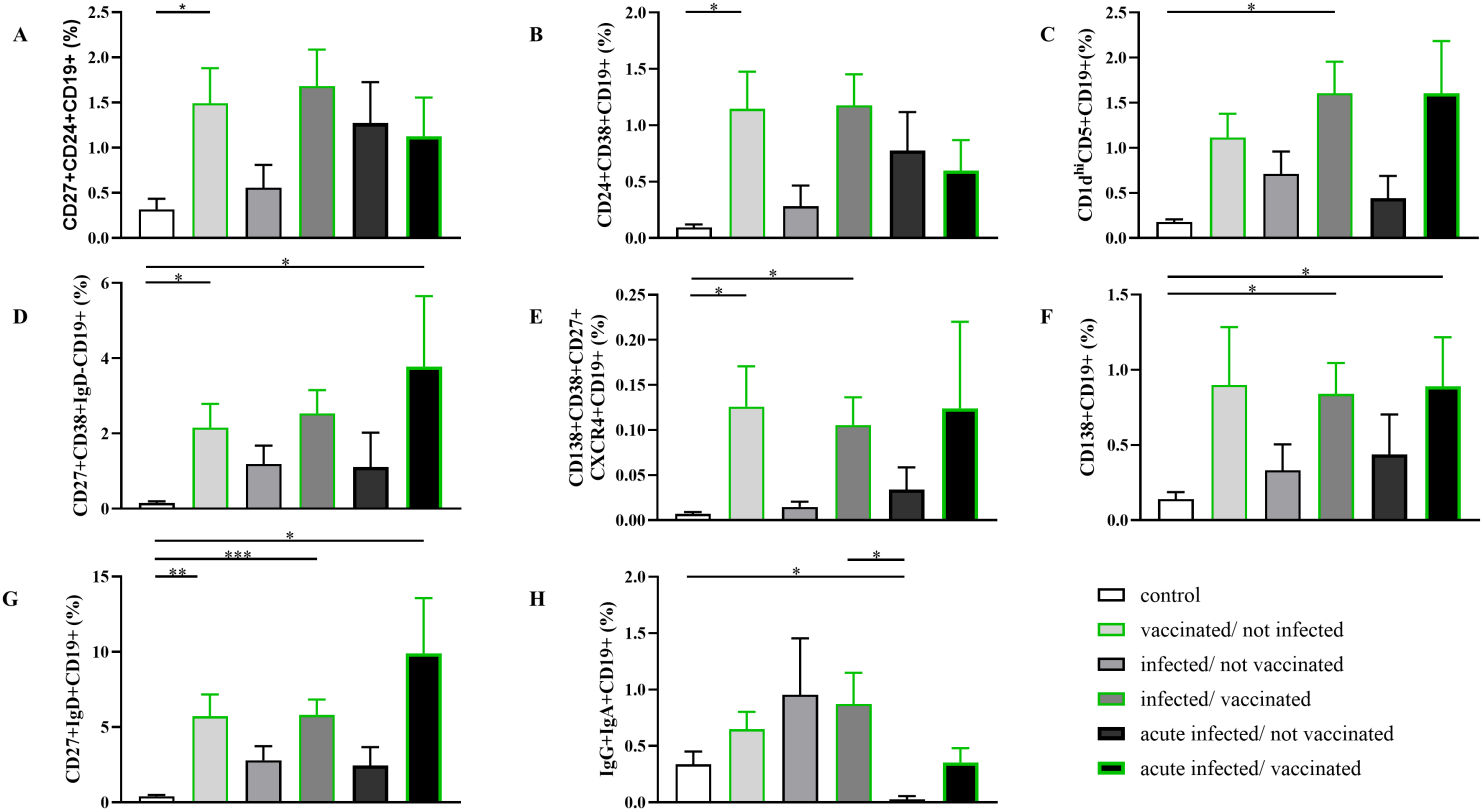
B cell populations in maternal blood before delivery in dependence of SARS-CoV-2 infection and vaccination. The determination of B cell populations was conducted through the utilization of flow cytometry. The following percentages are presented: CD27+CD24+CD19+ **(A)**, CD24+CD38+CD19+ **(B)** and CD1d^hi^CD5+CD19+ Breg cells **(C)**, CD27+CD38+IgD-CD19+ plasmablasts **(D)**, CD138+CD38+CD27+CXCR4+CD19+ short-living plasma cells **(E)**, CD138+CD19+ plasma cells **(F)**, CD27+IgD+CD19+ un-switched memory B cells **(G)**, and IgG+IgA+CD19+ class-switched plasma cells **(H)**. The data were obtained from 13 controls, 32 vaccinated/not infected, 23 infected/not vaccinated, 56 infected/vaccinated, 5 acute infected/not vaccinated and 10 acute infected/vaccinated patients. Data were analyzed by Shapiro-Wilk test and then Kruskal-Wallis test, followed by Dunn’s multiple comparisons test. Shown is the mean and SD.; *p<0.05, **p<0.01, ***p<0.001.

**Figure 3:**
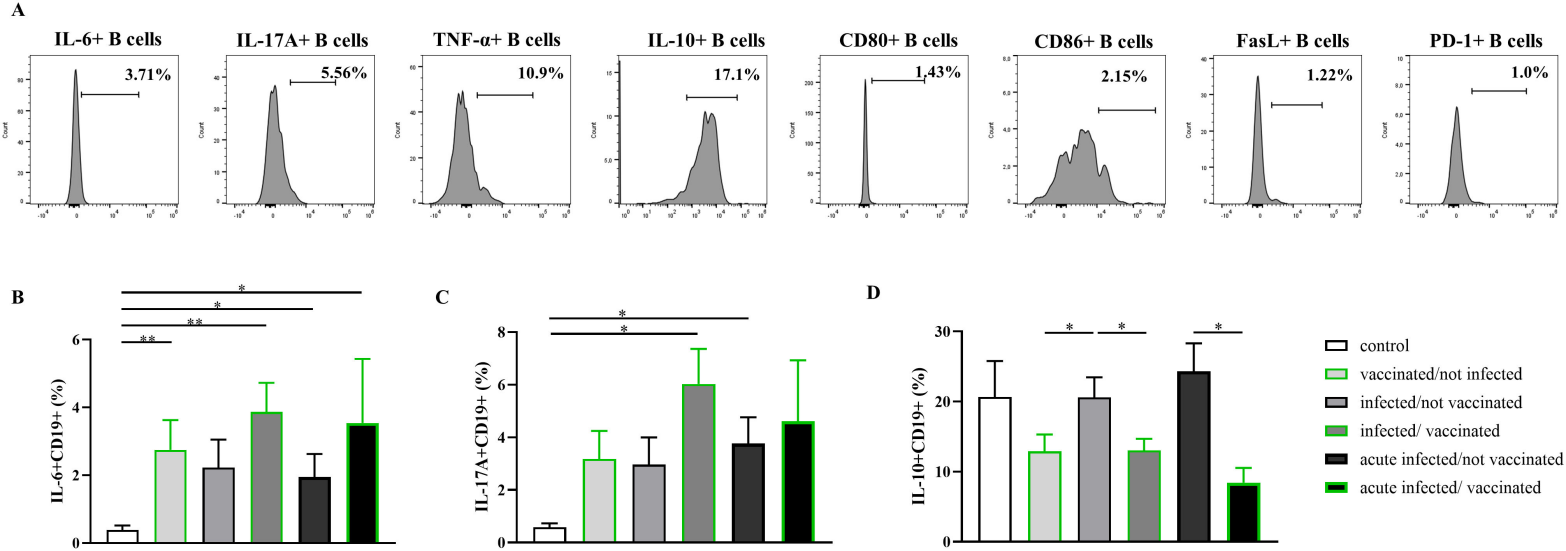
B cell-specific cytokine secretion in maternal blood before delivery, depending on SARS-CoV-2 infection and vaccination status. PBMCs from the 13 controls, 32 vaccinated/not infected, 23 infected/not vaccinated, 56 infected/vaccinated, 5 acute infected/not vaccinated and 10 acute infected/vaccinated patients were cultured for 5h with PMA, ionomycin and Brefeldin A, after which intracellular cytokine secretion was determined by flow cytometry. **(A)** The flow cytometry gating strategy for B cells expressing IL-6, IL-17A, TNF-α, IL-10, CD80, CD86, FasL or PD-1 was based on the gating: lymphocytes, single cells, living cells, and CD19+ B cells. The configuration of all gates was conducted in accordance with minus 1 controls. The percentages of IL-6+ CD19+ B cells **(B)**, IL-17A+ CD19+ B cells **(C)**, and IL-10+ CD19+ B cells **(D)** are shown. The data were analyzed using by Shapiro-Wilk test and then Kruskal–Wallis test, followed by Dunn’s multiple comparisons test. The mean and SD are shown; *p<0.05, **p<0.01.

A comprehensive analysis of maternal immune cell populations was conducted in relation to both SARS-CoV-2 infection and vaccination. The measurement of intracellular cytokines was conducted following a 5 h incubation of the PBMCs with PMA, ionomycin, and Brefeldin A. The gating strategy for the major B cell populations is shown in **Figure 1**. Alterations were determined:

- Breg cell populations CD27+CD24+CD19+ (**Figure 2A**), CD24+CD38+CD19+ (**Figure 2B**) and CD1d^hi^CD5+CD19+ (**Figure 2C**) increased following vaccination alone or combined with infection. The proportion of CD24+CD38+CD19+ (p= 0.0020), CD24+CD27+CD19+ (p= 0.0053) and CD27+CD38+ B cells (p= 0.0426) was found to be elevated in vaccinated/uninfected subjects in comparison to infected/unvaccinated patients.
- The frequency of plasmablasts (CD27+CD38+IgD-CD19+; **Figure 2D**), short-living plasma cells (CD138+CD38+CD27+CXCR4+CD19+, **Figure 2E**), plasma cells (CD138+CD19+, **Figure 2F**) and un-switched memory B cells (CD27+IgD+CD19+, **Figure 2G**) increased following vaccination, but a SARS-CoV-2 infection contributed only marginally. The percentage of CD27+IgD+CD19+ B cells was increased in vaccinated/uninfected patients compared to infected/unvaccinated women (p= 0.0390).
- Class-switched plasma cells (IgG+IgA+CD19+) were nearly absent in acute infected patients (**Figure 2H**).
- The gating strategy for B cell populations expressing cytokines and costimulatory molecules is shown in **Figure 3A**. Both vaccination and infection with SARS-CoV-2 induced IL-6 secreting B cells (**Figure 3B**) and IL-17A-secreting B cells (**Figure 3C**), whereas the levels of IL-10-secreting B cells (**Figure 3D**) were reduced. The frequency of IL-10+ B cells was lower in vaccinated/uninfected than in infected/unvaccinated patients (p= 0.0175).

In both control and infected/vaccinated women, IL-10+ B cells negatively correlated with IL-6+ B cells and a positively correlated with IFN-γ- and TNF-α-expressing B cells (**Supplementary Table 2B**). No significant differences were detected in total CD19+ B cells, naïve B cells (CD38+CD27-IgD+CD19+), CD27+CD38-CD19+ B memory cells and CD95+CD9+CD19+ B memory cells and FasL+CD19+ Killer B cells/Breg cells, as well as B cells expressing CD40, CD80, CD86, PD-1 or PD-L1 (**Supplementary Table 2**). Similarly, CD4+ T cells, CD4+ T cells expressing ICOS or CTLA-4, and CD25+Foxp3+CD4+ Treg cells showed no significant alterations (**Supplementary Table 3**).

### 3.4 Alterations in B cell populations following 72 h stimulation

**Figure 4:**
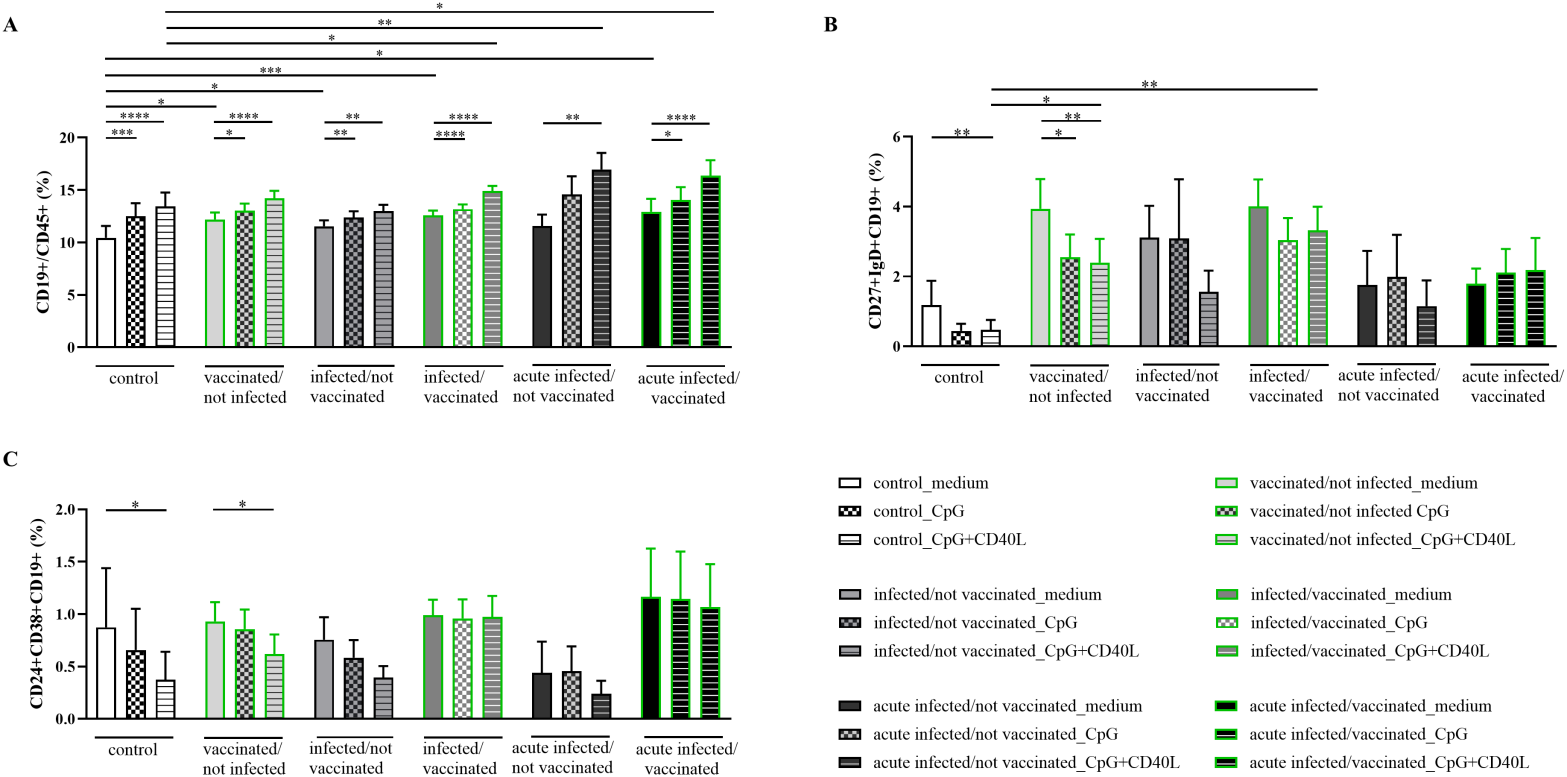
Maturation of maternal B cell populations following 72 h of stimulation with CpG and CD40L, depending on SARS-CoV-2 infection and vaccination. Maternal PBMCs from 13 control women, 32 vaccinated/not infected, 23 infected/not vaccinated, 56 infected/vaccinated, 5 acute infected/not vaccinated and 10 acute infected/vaccinated patients were stimulated with CpG or CpG and CD40L for 72 h or left untreated. The percentages of CD19+ B cells **(A)**, CD27+IgD+CD19+ USMBC **(B)** and CD24+CD38+CD19+ Breg cells **(C)** are shown. The analysis of the data was conducted using both the Shapiro-Wilk test and the Friedman’s test to evaluate variations within a patient group in response to stimulation (untreated compared to stimulated with CpG compared to stimulated with CpG+ CD40L), and the Kruskal–Wallis test to analyze differences between patient groups under the same treatment conditions (untreated, CpG or CpG+ CD40L between all groups). Shown is the mean and SD; *p<0.05; **p<0.01; ***p<0.001; ****p<0.0001.

Maternal PBMCs were stimulated with CpG or CpG and CD40L for 72 h, or left untreated. The analysis revealed inter-individual variations within the groups, as well as differences between the study groups following 72 h incubation with and without stimulation. The percentage of B cells was higher in previously vaccinated (uninfected, previously or acute infected) compared to control pregnant women at term (**Figure 3A**). The application of CpG and CpG+CD40L stimuli resulted in an augmentation of CD19+ B cell percentages within all groups, with significant differences between the groups only after CpG+CD40L stimulation. Here, B cell percentages were significantly higher in patients who were both infected and vaccinated or acutely infected, both vaccinated and unvaccinated, compared to control pregnant women (**Figure 4A**).

Several B cell populations showed alterations within individual groups upon treatment, including a reduction of CD27+IgD+CD19+ USMBC (**Figure 4B**) and CD24+CD38+CD19+ Breg cells (**Figure 4C**) in both controls and vaccinated/uninfected patients, and a decrease of CD24+CD27+CD19+ Breg cells in infected and vaccinated patients. None of these B cell populations were altered between vaccinated/uninfected and infected/unvaccinated patients. Following stimulation, the percentage of IgA+IgG+CD19+ CSPC declined in control women, while CD80 expression by B cells decreased in vaccinated but uninfected patients. Both controls and vaccinated/uninfected patients showed a reduction in FasL expression upon stimulation. The following B cell populations exhibited disparities between the groups in untreated as well as CpG and CpG+CD40L treatment conditions (**Supplementary Table 4**): CD138+CD38+CD27+CXCR4+CD19+ B cells, CD86+CD19+ B cells, CD95+CD9+CD19+ B memory cells and FasL+CD19+ B cells. Following the application of CpG+CD40L, a discrepancy was observed in the percentages of CD27+IgD+CD19+ and PD1+CD19+ B cells. A significant increase in the percentage of B cells expressing CD86, FasL and PD-1 was observed in vaccinated/uninfected patients compared to infected/unvaccinated patients, both in unstimulated samples and following stimulation with CpG alone or in combination with CD40L (**Supplementary Table 5**).

**Figure 5:**
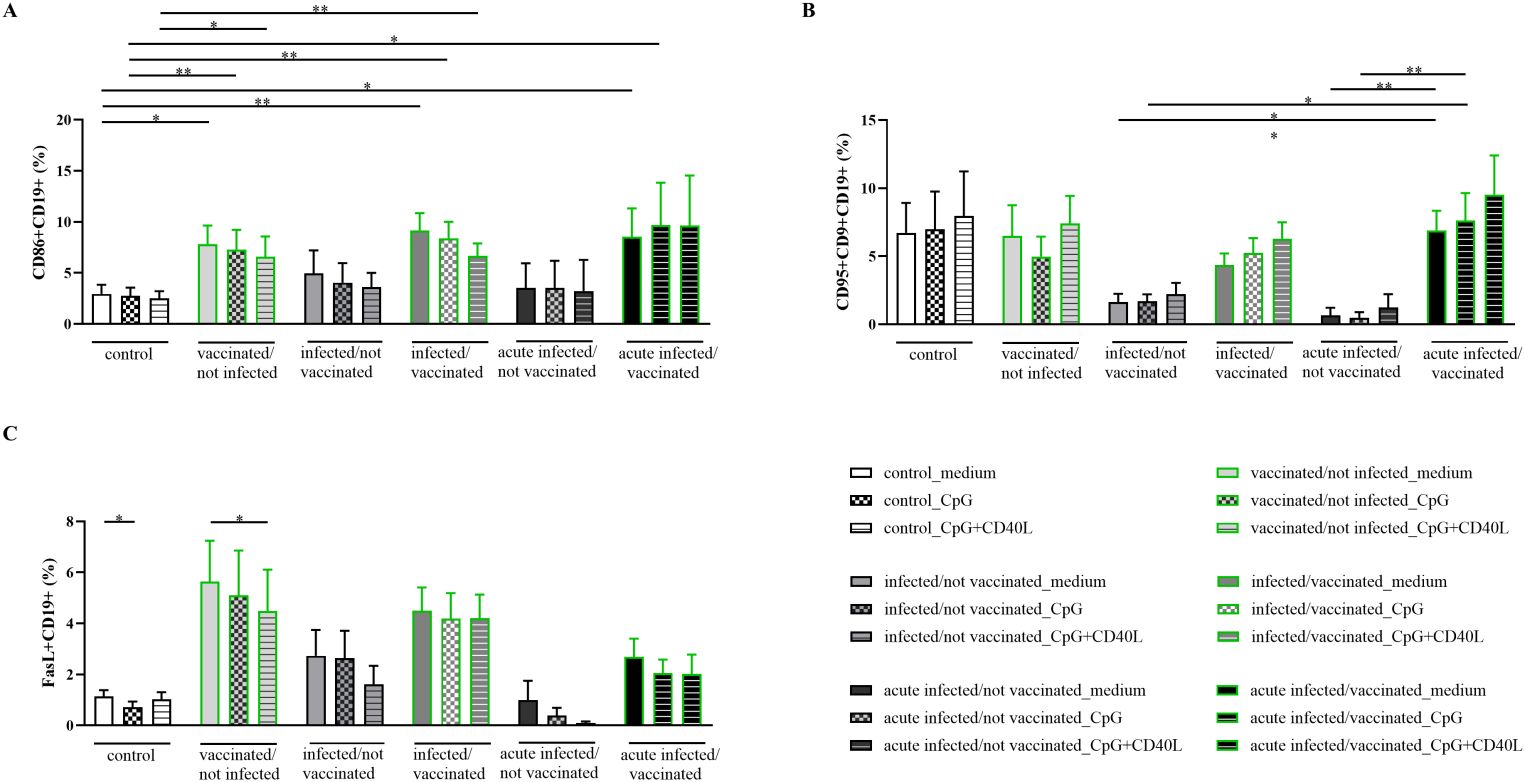
Maturation of B cell populations expressing co-stimulatory molecules following 72 h of stimulation with CpG and CD40L, depending on SARS-CoV-2 infection and vaccination. The percentage CD86+CD19+ B cells **(A)**, CD95+CD9+CD19+ MBC **(B)** and FasL+CD19+ B cells **(C)** were determined by flow cytometry following 72 h of stimulation with CpG or CpG+CD40L. Data were obtained from 13 control women, 32 vaccinated/not infected, 23 infected/not vaccinated, 56 infected/vaccinated, 5 acute infected/not vaccinated and 10 acute infected/vaccinated patients were analyzed using both the Shapiro-Wilk test and Friedman´s test (variations within a patient group due to stimulation) or Kruskal-Wallis test (differences between the patient group under the same treatment condition), shown is the mean and SD; *p<0.05; **p<0.01.

Furthermore, the frequency of IL-6+CD19+ B cells was found to be influenced by the patients’ vaccination and infection status under untreated conditions (**Supplementary Table 4**). B cells from vaccinated/uninfected patients in the medium control expressed elevated levels of IL-6, but diminished levels of IL-10, in contrast to those observed in infected, unvaccinated patients. The percentage of CD27+CD38+CD138+CXCR4+CD19+ and CD86+CD19+ B cells remained constant in response to stimulation with CpG or CpG+CD40L within each of the investigated groups. The expression of CD86 was lower in the controls and in patients with acute infection without vaccination (**Figure 5A**). In previous or acute cases of SARS-CoV-2 infection in unvaccinated patients, a decrease in the percentage of CD95+CD9+CD19+ B cells was observed. Stimulation did not alter the percentages of these cells in any group (**Figure 5B**). The expression of the CD95 ligand, FasL, was lowest in the control group and unvaccinated patients with acute infection. Following stimulation with CpG+CD40L, the percentage of FasL+CD19+ B cells decreased (**Figure 5C**). No differences were determined in the frequencies of CD38+CD27-IgD+CD19+, and CD38+CD27+IgD-CD19+ B cells CD1d^hi^CD5+CD19+ Breg cells, CD138+CD19+ B cells, CD40+CD19+ B cells and PD-L1+CD19+ B cells.

**Figure 6:**
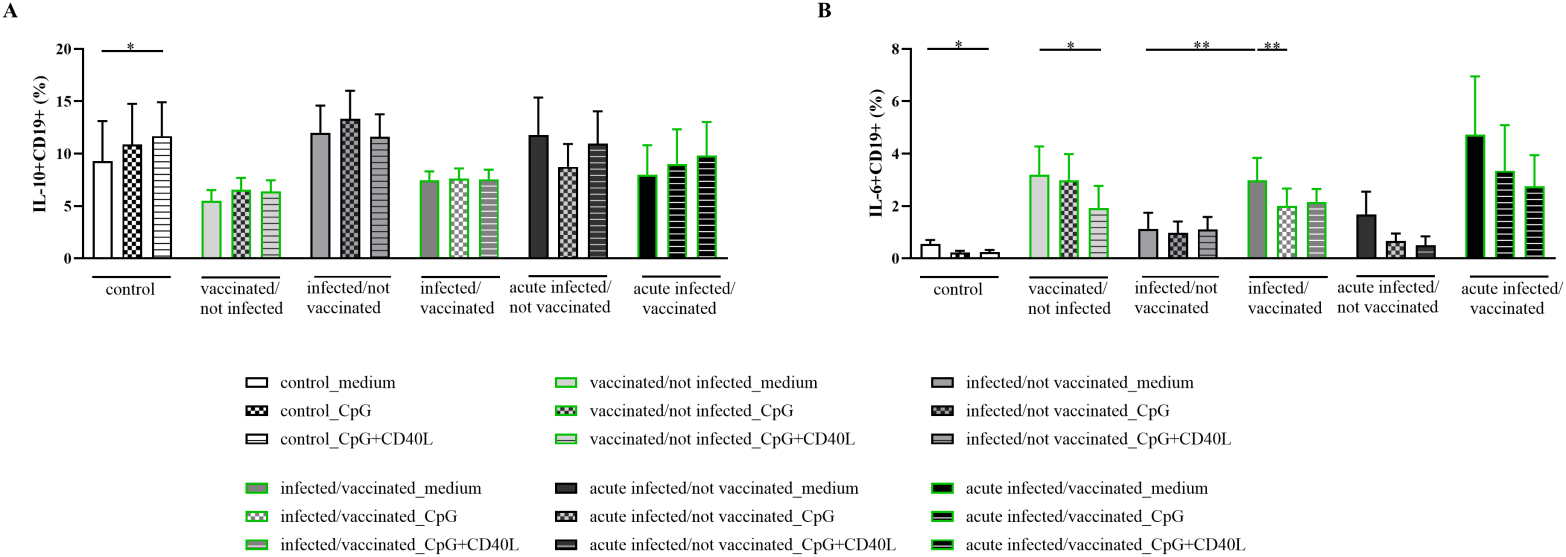
*Changes in the B cell-specific expression of IL-10 and IL-6 following stimulation.* Maternal PBMCs (13 control women, 32 vaccinated/not infected, 23 infected/not vaccinated, 56 infected/vaccinated, 5 acute infected/not vaccinated and 10 acute infected/vaccinated patients) were stimulated with CpG or CpG and CD40L for 72 h or left untreated. The percentage of IL-10+CD19+ B cells **(A)** and IL-6+CD19+ B cells **(B)** was determined by flow cytometry. Data were analyzed using both the Shapiro-Wilk test and Friedman´s test (variations within a patient group due to stimulation) or Kruskal-Wallis test (differences between the patient group under the same treatment condition), shown is the mean and SD; *p<0.05; **p<0.01.

Finally, stimulation with CpG+CD40L increased IL-10+ B cells in the control group (**Figure 6A**) and reduced the IFN-γ expression by B cells in vaccinated and infected patients, but reduced IFN-γ+CD19+ B cells in unvaccinated acutely infected patients. Controls and vaccinated patients with or without previous SARS-CoV-2-infection showed a stimulation-induced reduction of IL-6 expression (**Figure 6B**). The same tendency was found for IL-17A+ B cells in control and vaccinated/infected patients.

## 4 Discussion

Viral infections rapidly activate the innate immune response, which in turn triggers the adaptive immune response. This results in proliferation and differentiation of naive lymphocytes into T and B effector cells. Antibody-secreting B effector cells, also known as plasmablasts and plasma cells, contribute substantially to rapid and specific viral clearance.

Following infection with SARS-CoV-2, B cells release antibodies against spike and nucleocapsid proteins within a period of 5–15 days. The development of neutralizing antibodies against SARS-CoV-2 is typically straightforward, as it can be accomplished by numerous B cells with minimal or no requirement for affinity maturation. These antibodies are typically derived from naive B cells rather than from pre-existing cross-reactive memory B cells (17). SARS-CoV-2-specific B memory cells have been identified up to eight months following infection (18). The ongoing genetic diversification SARS-CoV-2 is driven by spike protein mutations (Alpha, Beta, Gamma, Delta, Omicron), which regulate receptor binding, immune evasion, and structural stability (19). Recently it was described that early variant-derived antibodies may inhibit activation of Omicron-specific naive B cells, explaining the significant immune imprinting observed in mRNA-vaccinated patients (20). However, it was also demonstrated that patients who had recovered from mild SARS-CoV-2 infections exhibited elevated levels of autoantibodies and activated B cells that respond to allergens (21, 22). One interesting observation are autoantibodies targeting G protein-coupled receptors that are found in both Long-COVID and preeclampsia, and both are associated with inflammation and immune tolerance (23–25). Furthermore, SARS-CoV-2 infection increased the risk for the development of preeclampsia (26). The mechanisms underpinning these effects remain to be fully elucidated, particularly in the context of pregnant women.

Pregnancy induces profound changes in the immune system. This phenomenon is necessary for the physiological adaptation of the female organism to pregnancy, as well as the immunological tolerance toward the fetus. Such adaptations are associated with increased susceptibility and frequent severe courses of infection in pregnant women, and may influence long-term specific immune memory. Initial SARS-CoV-2 variants caused higher rates of pregnancy complications such as premature birth, stillbirth and pre-eclampsia (27, 28). Consequently, SARS-CoV-2 vaccination was strongly recommended for pregnant women. Most pregnant women in our study received the Pfizer/BioNTech (Comirnaty) mRNA vaccine encoding the spike protein, which was the first SARS-CoV-2 vaccine to be successfully administered in pregnant women (29). In light of our preceding discoveries concerning the presence of elevated antibody titers against the spike and/or nucleocapsid protein in pregnant women at term following vaccination and infection (9), we hypothesized that there would be concomitant variations in the differentiation state of B cells, specifically an augmentation in the population of antibody-secreting B cells.

In non-pregnant individuals, a two-dose primary vaccine series demonstrated robust immunogenicity, eliciting a peak of circulating IgG- and IgA-secreting plasmablasts one week after the second immunization followed by a subsequent decline. Booster immunizations stimulate generation of germinal center B cells, which secrete antibodies characterized by elevated levels of somatic hypermutation, indicating a B memory cell origin and therefore a robust humoral immunity (30–33).

SARS-CoV-2 infection has been demonstrated to enhance maternal TNF-α and IL-10 plasma levels, while exerting minimal effects on IL-6 or IL-17A levels, regardless of the timing of infection during pregnancy or at delivery (34–36). These findings are consistent with our observations of only minor differences in maternal cytokines levels, although in our population, vaccinated women with acute SARS-CoV-2 infection demonstrated higher IL-6 and TNF-α concentrations compared to those who were acutely infected but not previously vaccinated. This outcome may be explained by predominantly mild course of disease in our cohort, in which “the cytokine storm” of severe SARS-CoV-2 infection was not observed.

Members of the TNF superfamily are key regulators of immune response. In healthy pregnancies, maternal serum levels of BAFF decreases, while APRIL levels remain stable throughout pregnancy (37). In a murine model, BAFF has been demonstrated to promote inflammatory responsiveness and increases susceptibility to inflammation-induced preterm birth. Conversely, APRIL exerts an opposing effect (38). At present, there is a paucity of data regarding the expression of APRIL, BAFF, and sCD40L during SARS-CoV-2 infections or vaccinations in pregnancy (38). In non-pregnant patients with COVID-19, increased levels of sCD40L, BAFF and APRIL were identified (39–41). Besides, patients diagnosed with Post-Covid-19 Vaccine Syndrome exhibited elevated sCD40L plasma levels, accompanied by modulated expression of pro-inflammatory cytokines and chemokines. Moreover, the presence of spike protein in monocytes was described approximately 250 days after vaccination (42). We detected elevated sCD40L levels in the serum of unvaccinated acutely SARS-CoV-2 infected patients. As sCD40L plays a central role in the linking of inflammatory and coagulation pathways, among others triggering tissue factor expression and contributing to clot stabilization, this finding indicates activation of the coagulation system, which appeared to be attenuated by vaccination. Furthermore, decreased levels of APRIL in unvaccinated mothers with a previous SARS-CoV-2 infection may negatively affect immune homeostasis, since APRIL is essential for B cell survival and the induction of IL-10-expressing Breg cells (43).

In the absence of stimulation, no changes total, naïve or memory B cells were observed following vaccination or infection in pregnant women at term. This contrasts with previous reports of reduced B cell frequencies in unvaccinated pregnant patients with moderate and severe COVID-19 (44, 45), likely reflecting the mild disease course in our cohort. Pregnant women recovered from COVID-19 demonstrated increased naive B cells (CD45+CD19+CD27-IgD+), accompanied by a concomitant decrease in B memory cell subsets (CD19+CD27+CD38-) (46). We observed a decline in B cells, encompassing both naïve and memory B cells, only in acutely infected unvaccinated mothers, consistent with COVID-19-associated lymphopenia (47). Nevertheless, the number of patients in this group is insufficient for the drawing of general conclusions.

An increase in B cell populations was detected, which have been described as exhibiting characteristics of Bregs cells, as well as in differentiated B cell subsets including plasmablasts and plasma cells in vaccinated pregnant women. This suggests effective stimulation of B cell maturation by SARS-CoV-2 mRNA vaccines. Notably, additional SARS-CoV-2 infection did not further enhance the differentiation of these B cell populations, underscoring the pivotal role of vaccination in promoting antibody-secreting B cell responses. Acute SARS-CoV-2 infection in pregnant women was associated with an increase in CD27+CD38+ antibody-secreting cells, comparable to that observed in non-pregnant women (48). Notwithstanding, vaccine-induced antibodies cells in pregnant women have been reported to exhibit reduced binding potency to the spike protein, diminished neutralizing potency and breadth, and a lower frequencies of spike-specific memory B compared with non-pregnant women (49). We observed an increase of un-switched B memory cells in vaccinated/uninfected patients compared to infected/unvaccinated women. A decline in the number of these cells was reported in patients with severe SARS-CoV-2 infection. The authors also described the production of IL-6 by un-switched memory B cells and the release of TNFα by switched memory B cells in response to CpG (50). Our previous studies identified an imbalance between inflammatory B cells and Breg cells in inflammation-associated preterm delivery and preeclampsia (51–53). In the present study, the healthy control group exhibited the lowest frequencies of IL-6 and IL-17A-secreting B cells compared to all other groups, alongside higher proportions of IL-10+ B cells compared to other study groups. While the maturation into IL-6 and IL-17A-secreting B cells was evident in all women after a vaccination or infection, IL-10+CD19+ B cells were less frequent in the vaccinated groups, despite increased proportions of several Breg cell populations. This suggests that possibility that vaccine- or infection-induced Breg cells may exert immunoregulatory effect via IL-10-independent mechanisms.

Stimulation with CpG alone primarily activates B memory cells, whereas co-stimulation with CD40L induces activation, proliferation and differentiation into CD24^hi^CD38^hi^CD19+ and IL-10+ B cells, CD27+CD38+CD19+ plasmablasts, plasma cells, and CD19+CD27+ memory cells (54–56). This treatment also induces the expression of co-stimulatory markers such as CD80, CD86, PD-1, and PD-L1 (57). Our results demonstrate that B cells from all patient groups proliferate in response to both CpG alone and CpG combined with CD40L. While no differences were observed following CpG stimulation alone, co-stimulation with CD40L revealed a lower proportion of cultured B cells in the control group compared to all other groups. This suggests that B cells from vaccinated and/or infected individuals may possess enhanced intrinsic proliferative capacity. Furthermore, vaccination or previous/acute SARS-CoV-2 infection does not appear to alter B cell response to T cell-independent stimuli, but may modulate responses requiring T cell-dependent signals such as CD40L.

CD40 expression is induced by B cell activation and is essential for the development of long-term immunity. We observed no alterations in CD40 expression following stimulation across groups. However, CD40 expression was significantly reduced in B cells from acutely infected vaccinated patients compared with their unvaccinated counterparts. CD40 signaling induces the expression of interferon regulatory factor 4 (IRF4), which activates nuclear factor-κB (NF-κB) and promotes plasma cell differentiation (58). Robust CD40 signaling plays a pivotal role in the initial immune response, specifically in the generation of B memory cells (59), which can subsequently differentiate into plasma cells. This process may be particularly relevant in unvaccinated pregnant women experiencing a primary encounter with SARS-CoV-2. Conversely, during recall responses, lower levels of CD40 are sufficient to drive the differentiation of germinal center B memory cells, as observed in vaccinated individuals with pre-existing immunity who can rapidly produce antibodies during acute infection.

We detected low frequencies of CD95+CD9+ B cells, which are specific markers for memory B cells (60), in unvaccinated patients with prior or acute SARS-CoV-2 infection. Although the precise function of CD95+ B memory cells remain unclear, these cells appear highly responsive to stimulation, suggesting an effector memory phenotype. Consistently, in our study, CD95+CD9+CD19+ B cells showed increased responsiveness to CpG and CD40L stimulation across all patient groups. CD95 (Fas receptor), expressed on activated lymphocytes, can support survival and proliferation, but can also induce apoptosis, thereby limiting inflammation depending on the context. It remains unclear whether the lower levels of CD95+CD9+ B cells in unvaccinated acutely infected women reflect SARS-CoV-2-induced inflammation and apoptosis, or alternatively, an expansion of this subset in previously vaccinated or infected individuals. The CD95 ligand (FasL) can be expressed by B cells following infection, has been associated with a regulatory phenotype through induction of apoptosis in T effector cells (61, 62). We found lower frequencies of FasL+ B cells in controls and unvaccinated acutely infected women, with a further decrease following stimulation. This indicates that vaccination or prior infection may promote expansion of this Breg population.

Data on CD86 expression in maternal B cells following SARS-CoV-2 infection or vaccination are limited. CpG stimulation induce CD86 expression, enabling B cells to function as antigen-presenting cells (63). Increased CD86 expression has been reported in non-pregnant patients with severe SARS-CoV-2 infection, and may indicate a disruption of peripheral tolerance (64). In our study, CD86+ B cell frequencies were elevated in most patient groups, except for controls and unvaccinated acutely infected patients, irrespective of stimulation conditions. Furthermore, stimulation reduced CD86 expression in several groups, suggesting that prior vaccination or infection induces sustained CD86 expression that may be downregulated upon secondary stimulation with CpG motifs, potentially limiting excessive immune activation.

Finally, we observed that B cell fate following CpG and CD40L stimulation was influenced by vaccination status and prior or acute SARS-CoV-2 exposure. These alterations affected the balance between inflammatory B effector cells and IL-10-secreting B cells. Low frequencies of IFN-γ-expressing B cells were present in controls and unvaccinated acutely infected patients but increased following stimulation. Elevated IFN-γ+ B cell proportions in all groups with prior exposure to SARS-CoV-2 antigens suggest the generation of antiviral effector B cells. Control subjects exhibited low frequencies of IL-17A+ B cells. Unvaccinated groups showed lower IL-6+ but higher IL-10+ B cells frequencies under untreated conditions, with a significant reduction in IL-6 and induction of IL-10 expression following stimulation observed only in the control group. We previously detected comparable patterns in women with preterm labor, characterized by elevated IL-6 levels in untreated B cells and reduced IL-10-producing B cells following stimulation with CpG and CD40L (53). These findings indicate a functional equilibrium between inflammatory and regulatory B cells in healthy term pregnancies, that can be disrupted by SARS-CoV-2 infection and vaccination.

The study has some limitations that should be considered. In view of the limited number of patients within the acute infected groups, the results obtained are exploratory in nature and necessitate a greater number of patients in order to confirm these initial observations. Whilst the present study has revealed a number of intriguing correlations, it has not sought to establish causal relationships. Consequently, the assertions concerning more extensive immune consequences or long-term effects have been qualified or qualified further.

A further limitation is the absence of assays that are specific to, or functional in relation to, SARS-CoV-2. This limits the ability to draw conclusions about a primary or booster B cell response, and in particular about antiviral immunity. However, the majority of women were infected in the second and third trimester, and received a booster vaccination in the first and second trimester. The presence of antibodies against SARS-CoV-2 in patients, contingent on their infection and vaccination status, suggests a probable association.

Taken together, our data suggest that exposure to SARS-CoV-2 antigens, either through vaccination or infection, has a profound impact on the maternal immune system. Further studies are required to elucidate the long-term implications of these alterations for maternal and offspring health.

## Data Availability

The raw data supporting the conclusions of this article will be made available by the authors, without undue reservation.

## Supplementary material

Supplementary Table 1 The following is a list of the utilized antibodies in the present study, including the relevant order numbers and the respective companies.

Supplementary Table 2 Spearman correlation analyses were conducted on maternal serum **(A)** and on IL-10+ B cells and IL-6-, IFN-γ-, and TNF-α-expressing B cells **(B)**.

Supplementary Table 3 Shown are the mean and SD values of the percentages of total CD19+ B cells, CD38+CD27-IgD+CD19+ naïve B cells, CD27+CD38-CD19+ B memory cells, CD95+CD9+CD19+ memory B cells, FasL+CD19+ Killer B cells/Breg cells, B cells expressing CD40, CD80, CD86, PD-1 or PD-L1, IFN-γ+CD19+ and TNF-α+CD19+ B cells. Furthermore, the proportion of CD4+ T cells, ICOS+CD4+, CTLA-4+CD4+ T cells and CD25+Foxp3+CD4+ Treg cells were shown.

Supplementary Table 4 Shown are the mean and SD values of the percentages of total CD19+ B cells, CD38+CD27-IgD+CD19+ naïve B cells, CD1d^hi^CD5+CD19+ and CD24+CD27+CD19+ Breg cells, CD24+CD38+CD19+ transitional B cells/Breg cells, CD27+CD38+CD138+CXCR4+CD19+ short-living plasma cells, CD27+IgD+CD19+ unswitched memory B cells, CD38+CD27+IgD-CD19+ plasmablasts, CD138+CD19+ plasma cells, CD27+CD38-CD19+ B memory cells, CD95+CD9+CD19+ B memory cells and FasL+CD19+ Killer B cells/Breg cells and B cells expressing CD40, CD80, CD86, PD-1 or PD-L1, IFN-γ, IL-6, IL-17A, IL-10 and TNF-α. Friedman analysis revealed variations within the study groups in response to stimulation, and Kruskal-Wallis tests were used to identify differences between the individual groups, both untreated and following stimulation.

Supplementary Table 5 **(A)** The results of the Mann-Whitney tests are presented here, showing the percentages of total CD38+CD27-IgD+CD19+ naïve B cells, CD27+CD38+CD138+CXCR4+CD19+ short-living plasma cells, CD95+CD9+CD19+ memory B cells and FasL+CD19+ Killer B cells/Breg cells and B cells expressing CD40, CD86, PD-1, IL-6 or TNF-α. These results are shown between vaccinated/not infected vs. infected/not vaccinated, both untreated and following stimulation. **(B)** The results of the Kruskal-Wallis tests are displayed, showing the percentages of total CD38+CD27-IgD+CD19+ naïve B cells, CD27+CD38+CD138+CXCR4+CD19+ short-living plasma cells, CD95+CD9+CD19+ memory B cells and FasL+CD19+ Killer B cells/Breg cells and B cells expressing CD40, CD86, PD-1, IL-6 or TNF-α. These results are shown between vaccinated/not infected vs. infected/not vaccinated vs. infected/vaccinated, both untreated and following stimulation.
